# *E*^2^*JSL*: Energy Efficient Joint Time Synchronization and Localization Algorithm Using Ray Tracing Model

**DOI:** 10.3390/s20247222

**Published:** 2020-12-17

**Authors:** Rehan Shams, Pablo Otero, Muhammad Aamir, Fozia Hanif

**Affiliations:** 1 Department of Telecommunication, Sir Syed University of Engineering and Technology, Karachi 75300, Pakistan; muaamir05@gmail.com or; 2 Department of Telecommunication, Universidad De Malaga, E-29071 Málaga, Spain; pablo.otero@uma.es; 3 Department of Mathematics, University of Karachi, Karachi 75300, Pakistan

**Keywords:** localization, time synchronization, energy efficiency, ray tracing, stratification effect

## Abstract

In underwater wireless sensor networks (UWSNs), localization and time synchronization are vital services that have been tackled independently. By combining localization and time synchronization, could save nodes energy and improve accuracy jointly. Therefore, it is of great significance to study joint synchronization and localization of underwater sensors with low energy consumption. In this paper, we propose the energy-efficient joint framework of localization and time synchronization, in which the stratification effect is considered by using a ray-tracing approach. Based on Snell’s law, ray tracing is applied to compensate for the variation of sound speed, this is one of the contributions of this article. Another contribution of this article is the iteration process which is used to improve the accuracy of localization and time synchronization. Simulation results show that the proposed joint approach outperforms the existing approaches in both energy efficiency and accuracy. This study also calculates Cramer-Rao lower bound to prove the convergence of the proposed technique along with the calculation of complexity of the proposed algorithm to show that the provided study takes less running time compared to the existing techniques.

## 1. Introduction

Many applications are associated with underwater environments such as surveillance, ocean monitoring, and disaster mitigation to measure the level of the sea due to the melting process of ice.

Acoustic communication is the process of receiving and sending messages by using sound waves in an underwater environment. The underwater architecture usually consists of sensors and vehicles that deploy in some area of the seafloor to record the data at a fixed location. The collection of underwater information can be possible due to randomly placed underwater sensors that collect some important hydrologic data for example pressure, temperature, and salinity. However, it is important to recover these deployed sensors after the completion of the task. Underwater sensor nodes are mostly in moving positions because of the sea flow and other creatures after their deployment. It is very important in many applications that the location of sensors is known, otherwise, the collected information is meaningless and since the localization is depending upon synchronization then it is very useful to perform both localization and time synchronization within the same scenario. The major disadvantage of traditional local approaches is a lack of communication, and the recorded data could be destroyed in the case of any failure. A wide variety of applications are supported by underwater sensors such as river and sea pollution discovery, aquatic surveillance, monitoring, commercial exploit the aquatic environment oceanographic data. UWSNs applications require two types of services which are depending upon each other. For example, time division multiple access (TDMA) is an important medium access control (MAC) protocol that often requires accurate time synchronization between sensor nodes. The information exchange process about the local clock drift is the most common procedure in UWSNs. An accurate localization technique is required to monitor the underwater surroundings because the collected information is only beneficial if the nodes are localized up to certain accuracy, otherwise, the gathered information is meaningless. Localization is also very important for surveillance applications.

Long-range missions can be possibly done by long endurance “autonomous underwater vehicle” AUVs, for example, underwater gliders which are energy efficient. These vessels mostly spend their time navigating along a straight course which is controlled by an inertial navigation system or electronic compass during this whole long-term mission. However, these underwater gliders don’t aware of their position and path that make them affected by ocean currents and waves. Today, we use such vehicles which are the only autonomous underwater platforms that can collect the ocean information without any local assistance for several months. Hence, they are considered a powerful tool for the improvement of ocean knowledge.

Like localization, synchronization is a fundamental problem in distributed sensor networks especially since the timestamp is an important factor while sensing the data.

Furthermore, underwater sensor nodes have limited energy resources, which has a substantial impact on the lifetime of the network. Therefore, an energy self-sufficient UWSN is essential to maximize the lifetime of the network.

In comparison with traditional wireless sensor networks (WSN), the underwater environment is based on anchor nodes with known locations and synchronized clock, also, there are ordinary nodes whose location is unknown, and their clocks need to be synchronized. The process of finding the location and synchronization is mainly associated with message exchange between sensors and anchor nodes, but in most cases, are handled separately [1,2]. Because it was initially assumed for underwater localization, that all acoustic signals travel with the speed of 1500 m/s, due to this, methods like [3] provide a high estimation error. However, later, this error was improved by taking the fact that sound speed should be considered as an unknown perimeter.

Usually, this problem is simultaneously solved by synchronizing the clock first then locating the position of unknown sensors. Because of the poor efficiency of handling this problem independently, joint efforts have started to get good results due to the dependency of these two processes of localization and time synchronization. In UWSNs most of the techniques of accurate localization are based on the measurement of time of arrival (ToA) and their clock synchronization between the nodes, in contrast to this process of time synchronization takes advantage of the location information which can be further use in calculating the propagation delay. The message exchange between anchor and sensors has got both the information of location and clock information. Based on this information, the localization of sensor nodes and clock information can be calculated simultaneously with the help of a single sort of message, which ultimately reduces the energy.

Due to all the above-mentioned facts that explain the dependency of localization and time synchronization on each other, we can say that solving these two problems in a single framework, not only gives accuracy but also provides energy efficiency.

Besides the most important challenges of underwater that is, localization and time synchronization, consideration of the depth-dependent SSP (sound speed profile) which does not travel in a straight line and dependent upon the pressure, temperature, and salinity [4] is another main challenge, because in a real UWSNs environment, sound waves do not travel in a straight line. Propagation path bents and the node distance between the nodes do not travel by an acoustic wave. This phenomenon is called the stratification effect [5,6]. Based on the ray tracing approach, the object of this study is to apply the joint synchronization and localization five-phase algorithm called “Energy Efficient Joint Time Synchronization and Localization” using ray tracing model $E^{2}JSL$ with the consideration of the stratification effect. To handle the stratification effect, first, we use to introduce the ray-tracing model that develops a framework in which the stratification effect, clock drift, and sensor node location are estimated. The algorithm starts with a message exchange procedure between the anchor and sensor node. Ordinary sensor nodes that need to be localized are moving with depth information whereas the static anchor node has got all the information such as its location and time. A Comparison with different approaches has been made to prove the validity of the proposed technique in the simulation section with the estimation of the Cramer-Rao lower bound (CRLB) for the convergence analysis. Also, the complexity of the proposed algorithm is calculated to show that the provided study takes less running time compared to the existing techniques. As far as the limitation of the provided technique is concerned, provided study will not work in a multi-hop environment due to the one-way message exchange condition, but by making some additional assumptions, the proposed algorithm will work for the multi-hop environment for a large scale underwater localization and time synchronization.

The proposed research work is based on the following sections. Section 1 gives the introduction, Section 2 provides some information about the challenges in underwater sensors, Section 3 shows the comprehensive related work, Section 4 indicates that how the ray-tracing approach is beneficial for the proposed work. Section 5 gives the network assumptions followed by Section 5.1 which provides the proposed ray-tracing model, which is further extended in subsections Section 5.2 with the calculation of direct distance traveled by the ray. Section 6 calculates the pairwise localization and time synchronization, wherein Section 6.1 shows that how the proposed model works iteratively to get the accuracy, Section 6.2 gives the summary of each phase. Section 7 calculates the Cramer-Rao lower bound for the proposed algorithm along with the complexity computation in Section 7.1. Section 8 shows the simulation settings, while Section 9 provides the performance evaluators, and further, these sections also give the comparison for the proposed model with previously developed models by using different parameters. After this, we have a conclusion and then we have the references.

## 2. Challenges in Under Water Sensors Localization and Time Synchronization

Various issues and challenges are associated with the underwater environment as far as localization and time synchronization is concerned, some of them are as follows:

The most common challenge in UWSNs is node deployment which is not only difficult but also very costly in the deep-sea environment. Secondly, drifting of modes due to the different activities and current of the underwater environment. If a node displaces from its position, then it is not possible to estimate its accurate location. In third place, it’s a signal strength that is easily affected by many factors such as external noise, multipath propagation attenuation, and Doppler shift. Fourthly, it is a sound speed variation, which has taken to be constant in most of the localization techniques, but in actual it varies due to temperature, pressure, and salinity. Variation in any of these above-mentioned factors can cause a change in sound speed. Due to the high propagation delay, the speed of sound is five-time lower than radio waves. UWSNs face constraints like limited bandwidth, high propagation delay, 3D topology, and power constraints. Radio and optical waves are not feasible for communication at each point of the ocean. Also, the transmission range [7], frequency, and the data rate are less, and the bit error rate [8] is high. All the above-mentioned factors heavily affect the localization process, which means, to achieve accurate localization, these factors should be handled.

Time Synchronization is another main challenge that needs to be calculated simultaneously with localization. synchronization is mostly ignored in many localization schemes, most of the researchers assumed that nodes are already synchronized. Since radio signals cannot travel underwater therefore it is difficult to achieve synchronization due to long propagation delay and variable sound speed, also GPS service is not available underwater. Challenges related to synchronization are many, but the most common is the cost of underwater devices which are not easily available in the market, as all these expensive devices need protection and high-power electricity to transfer the underwater data. Another main challenge in UWSNs is that the standard underwater transducer does not receive and send messages at the same time, which is why it is important to have the information about the sending and receiving time of messages during the collection of data in UWSNs.

## 3. Related Work

Many researchers have made their efforts to address this problem, but all these previously developed techniques have different drawbacks along with their strengths. Here, we are discussing the already known algorithms related to this address problem. Various localization algorithms are discussed for the localization of UNSNs in [9]. In which the authors have divided the localization techniques into three categories such as, stationary localization algorithms, mobile localization algorithms, and hybrid algorithms. Although these algorithms have their individual strength, mostly they are unable to handle the stratification effect, which means that sound waves do not travel in a straight line underwater. Similarly, different time synchronization algorithms are given by [10] which are not so accurate due to the negligence of propagation delay underwater. As considered “joint time synchronization and localization design for mobile underwater sensor networks” (JSL) was the first effort which is made by the researcher for the joint procedure of localization and synchronization with the consideration of the stratification effect. This joint technique was given by [11] which only compensated the stratification effect in the underwater medium along with this assumption that sound waves travel in a straight line. This method jointly improves the accuracy by using the advanced tracking algorithm and to increase the accuracy in mobile node localization, interactive multiple models (IMM) are implemented. Snell’s law has been used by the author to calculate the stratification effects, but this method gives low accuracy because of the assumption that sound wave travels in a straight line. JSL localized and synchronizes the nodes with compensation of stratification effect which works in four phases. Phase I is the partitioning of 3D underwater sensor networks in a short latency, phase II performs the localization, phase III gives the time synchronization, and finally, phase IV is the refinement procedure. Another method was proposed by [12] that applies atomic multilateration and iterative multilateration method to localize and synchronize the sensors node. This method initially makes the partitioned of 3D space, but this process gives poor synchronization since it does not consider the clock skew during synchronization and stratification effect. In the network architecture, in this method, there are four or more anchor nodes with a known location on the water surface that sends their time and location information to ordinary sensor nodes to perform the process of localization and time synchronization. After getting messages from five anchor nodes, sensor nodes can estimate their location and time information. Due to the ignorance of mobility and clock skewness, these techniques give low efficiency with the consideration of constant sound speed.

A sequential time synchronization and localization (STSL) was given by [13] which showed better results. However, it (STSL) did not consider the stratification effect which gives low accuracy in localization, but it improves synchronization by taking variable sound speed in a dynamic underwater environment. In STSL each node is equipped with directional navigation systems for the accurate estimation of short-term motion which makes this procedure very costly to implement. Although this procedure gives better localization accuracy with the consideration of mobility of the sensor as well as anchor nodes. Another drawback of STSL is that the anchor nodes are not considered as time-synchronized during this process. The main point of this scheme is that it functions with only one anchor node. It also performs a self-evaluation of localization accuracy, by estimating the propagation speed and checking its validity, relying on known model boundaries for it. Although this method is use full for the estimation of nodes that possess short-term motion for the long-term mobile nodes this method is quite expensive. Another effort was made for a joint procedure of localization and time synchronization based on the least square method that consists of five phases that iteratively compensate for the stratification effect [14]. Phase I is the message exchange procedure between the anchors and sensor node. Phase II estimates the position and clock parameters, phase III estimates the stratification effect compensation, phase IV is the calculation of sound velocity and finally, phase V performs the iteration process. This approach is based on the assumption that rays travel in a straight line which radially decreases its efficiency, but the iterative procedure supports improving the accuracy of localization and time synchronization. In this joint effort, the stratification effect is calculated along with the sound effect with the help of an iterative method, but this paper did not calculate the continuous deflection of sound wave angle it only measures the initial angle of deflection of sound waves. Z. Gong et al. [15] has given a quite successful joint technique of localization and synchronization in UWSNs which is considered the stratification effect and to solve this problem maximum likelihood estimator has derived. Like STSL, this procedure also works in two-phases, during the first phase initial clock skew is ignored due to its small value. Then LS estimator is employed to get localized and synchronized by transforming the nonlinear equations into a linear one. With the help of another least square (LS) estimator, the coarse estimation is further refined in the second phase. However, this method is not economical and has poor scalability and like other joint techniques, this method did not consider the stratification effect. This method works on the concept of iteration that gradually improves efficiency. This method can’t provide any guarantee for the achievement of accurate results. As defined earlier that the calculation of propagation delay is very essential for time synchronization, this joint effort called “Energy-Efficient Time Synchronization and Localization Algorithm for Underwater Acoustic Sensor Networks” TSLA [16] ignores both the stratification effect and propagation delay although the provided technique is energy efficient due to the one-way packets exchange and node are in duty cycle which make this technique energy efficient. Low cost distributed networked localization and time synchronization were achieved by [17] and are robust to noisy measurement. Implemented in a testbed based on Teledyne Benthos Telesonar SM-975 underwater modems, these solutions possess no additional overhead. Later, this technique was tested extensively in Lake LaSalle at the University at Buffalo. This method consists of four phases, with localization first, and the output of this process is the input of synchronization for each round of message exchange. This technique fails to give accuracy due to the consideration that sound wave travels in a straight line. Also, this technique only works for the low energy budget.

A method called “Ameliorated Joint Synchronization and Localization for 3D Underwater Sensor Networks” (AJSL) [18] uses iterative weight least square estimator for 3D underwater sensor networks to ameliorated joint localization and synchronization. This method uses the benefit of projection procedure which inherent relationship between localization and synchronization with known depth. As far as the drawback of this technique is concerned this process is less energy efficient which uses an iterative least square method that radially increases the computational complexity. Also, this technique does not compensate for the stratification effect.

The work proposed in [19] is a unified framework to execute synchronization and localization simultaneously by considering the stratification effect which is the most essential factor to get accuracy in time the process of synchronization and localization. In this method, considered the stratification effect of the underwater medium has been modeled with the help of a ray-tracing approach. The maximum likelihood (ML) estimator is derived, which works as a nonlinear and nonconvex operator, that is why the Gauss–Newton algorithm has applied iteratively to solve the original nonconvex ML problem. This approach is quite accurate and provides better accuracy. Due to this, we have compared our proposed algorithm with this technique. Another sequential algorithm has also been presented in [20] which is a joint effort with the consideration of unknown sound speed. This method is a sequence of two linear estimations with movable nodes. However, like other abovementioned techniques, this procedure relying on the assumption that nodes are equipped with self-navigation systems therefore it can give accuracy for short periods. This method offers a heuristic solution for joint localization and time synchronization problems. All the above-mentioned algorithms jointly localized and synchronized the underwater sensors, but all these methods have some loopholes towards the accuracy. Therefore, the effort is required that jointly localize and synchronize the underwater sensors with low energy consumption and less computational complexity. The object of this study is to propose a five-phase method to jointly localize and time-synchronize the underwater sensor which is energy efficient and gives less computational complexity by using a ray-tracing approach. The reason behind choosing this topic is that very few researchers have addressed this problem with the consideration of stratification effects and propagation delay. We are compensating for the stratification effect by using a ray-tracing model that gives low complexity. Experimental results prove that our proposed approach outperforms as compare to the existing algorithms STSL, AJSL, and GN-JSL and takes less running time compared to these traditional techniques.

## 4. Significance of Ray Tracing Approach

As described above, ray tracing is a very useful approach which was previously used in field- strength simulation and transmission loss model that possess complex environment. It can be directly used for integration by using standard numerical integrators like the Runge-Kutta method. To develop the model for sound propagation in shallow water combination of the X-ray model and fourth-order Runge Kutta integration method can be used for ray tracing to full-field modeling of interactions with seabed [21]. A RAYSON model named as Ray in French is RAY and sound is SON is used for underwater communication of time-varying channels which is modeled with the integration of static parameters and variable parameters [22]. For the ray tracing of the ocean environment with irregular bottoms implementation, Hamilton’s equations are used called versatile three-dimensional “Hamiltonian acoustic ray-tracing program for ocean” (HARPO) [23]. Another approach in which the domain of the problem is divided into different subdomains. And each subdomain is fitted by the sound speed in a simple form whose analytic solution is possible [24]. One of the earliest forms of Gaussian beam propagation in the sonar community Porter with BELLHOP is introduced by Porter and Bucker. The Gaussian curve is sometimes calling a bell-shaped curve. So, BELLHOP is a hopping bell-shaped curve through the field. In this process, initial bandwidth and curvature are given to the Gaussian beam at the source point, which can expand and contract after going away from the source point [25,26]. Another model called “Comprehensive Acoustic Simulation System/Gaussian Ray Bundle” (CASS/GRAB) related to ray tracing is used by the Gaussian ray bundle instead of Gaussian beams [27,28], it has many applications such as Sonar Simulations in Applied Physics Laboratories of Washington Modular Ocean Data Assimilation System [29,30].

## 5. System Model and Proposed Technique

### 5.1. System Architecture and Assumptions

In this section, we define our network architecture with some assumptions.

**Figure 1:**
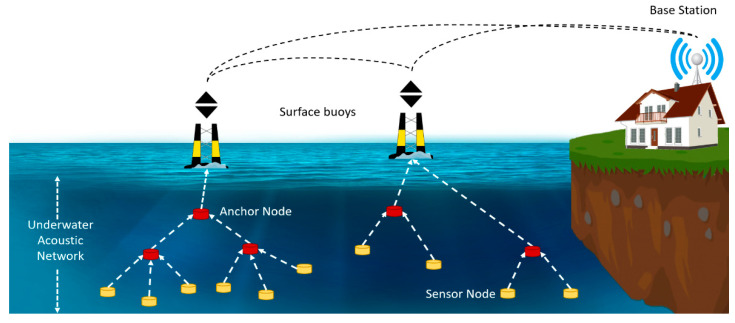
System architecture for underwater acoustic networks.

Generally, underwater sensor architecture consists of three important features: buoys, anchor nodes and, sensor nodes. As indicated earlier each anchor node is already synchronized and got all the information about their location, which could also work as a reference node. Whereas buoys can get their time information and location with the help of GPS, as each buoy is equipped with GPS. Lastly, we have the sensor nodes whose location and time information needs to be estimated by direct communication with anchor nodes. Here, we assume that each sensor node possesses three main features: sensing, communication, and computation. All these nodes are randomly distributed and collect information about the stochastic events as given by Figure 1. According to the proposed scenario, only one node will be synchronized and localized by using four anchor nodes. As indicated above all these anchor nodes know their positions and timings. Suppose we have *i*th anchor $A_{i}$ having an initial skew α and initial offset β which is located at [*x*, *y*, *z*]. The ordinary sensor node that needs to be synchronized is denoted by B and its location is $\left[x^{s},\;y^{s},\;z^{s}\right]$, which will need to know but the $z^{s}$ is known along with the skew and offset. The initial procedure starts with range-based localization that gives high accuracy as compare to range-free localization. It initially estimates the distance and angle by using angle of arrival (AoA), time of arrival (ToA), receiver signal strength (RSS), or from the network connectivity, then it employs triangulation or multilateration for the transformation of ranges into coordinates (least square method). The anchor node sends M rounds of messages to the sensor node, during the mth round of messages anchor $A_{i}$ sends a message at time $\eta_{A}$ which is received by the sensor node at time $T_{A}$ then sensor node B sends the acknowledgment message to the anchor node $A_{i}$ at time $T_{B}$ which is received by the anchor node at time $\eta_{B}$. In this way, node B collects a set of timestamp information $\left[\eta_{A},\;T_{A},{\;\eta }_{B},\;T_{B}\right]$ after M rounds of messages. 

### 5.2. Proposed Ray Tracing Approach

The proposed study will address the stratification effect by using the ray-tracing approach. The acoustic propagation is usually treated with a ray theory approach and considered as a valid approximation for the underwater environment.

By using the ray-tracing theory, we want to formulate the propagation model. To drive the equation for the time of flight, which is the total time taken by the acoustic ray and the change in flat range as the ray travels, we have used the ray parameter and ray geometry concepts.

We take the initial value of ray parameter ξ as,

(1) $$\xi =\frac{cos\theta_{o}}{c_{o}}$$

where $c_{o}$ is initial sound speed of the ray. From Snell’s law, the parameter is constant at each point along the ray.

$$c_{o}cos\theta \left(z\right)=c\left(z\right)cos\theta_{o}$$

$$\xi =\frac{cos\theta_{o}}{c_{o}}=\frac{cos\theta \left(z\right)}{c\left(z\right)}$$

**Figure 2:**
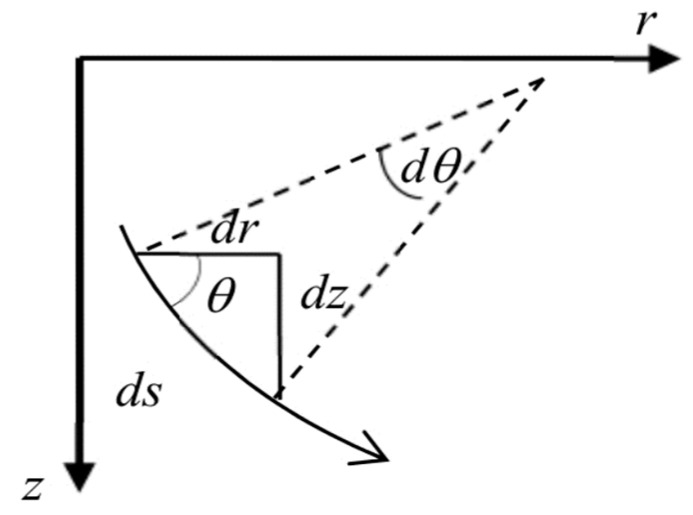
Ray traveling approach.

According to Figure 2, we consider a small ray segment in the two-dimensional system with d*s*, d*r*, and d*z* as horizontal and vertical components of the ray segment; *θ* is the launching angle of the ray.

Consider the medium having the ray path curve with a depth depending on the speed of sound, and the ray path will take the shape of an arc from a circle when this variation is of the form of constant slope, with a radius of curvature *R* in Equation (2).

(2) $$R\left(z\right)=-\frac{1}{\xi g\left(z\right)}$$

where ξ is the ray parameter defined earlier in Equation (1) and the sound speed gradient is *g*(*z*) which is given by Equation (3),

(3) $$g\left(z\right)=\frac{dc\left(z\right)}{dz}$$

We need to find the initial launching angle $“\theta_{o}”$ and the total time traveled by the ray “t” while starting with positions of the anchor and target sensor in (*r*,*z*) coordinates, the sound speed relationship in a medium where the gradient is linear between a point 1 $\left(r_{1},z_{1}\right)$ and a point 2 $\left(r_{2},z_{2}\right)$ is given as,

(4) $$c\left(z_{i+1}\right)=c\left(z_{i}\right)+g_{i}\left(z_{i+1}-z_{i}\right)$$

where $z_{i}$ and $g_{i}$ in the above Equation (4) are the depth of sound speed c and gradient in the segment respectively. Note that within each of these layers, sound speed has a constant gradient, the ray in each layer follows a circular arc pattern and the arc’s radius of curvature $R_{i}\left(z\right)$ is given (3) which is based on the local sound speed gradient and the ray parameter ξ, given in Equation (1) and Equation (4) respectively.

In this study, we use the sound velocity profile list in tabular form [31] and with the help of this data list between sound *Velocity* with the relative depth, we have calculated the regression equation [32] is given by Equation (5),

(5) $$Velocity=1522.67664+4.5022321\times 10^{-3}\times \left(depth\right)$$

In Equation (5) the unit of velocity is in m/s and the unit of depth is in m. We want to derive the equations for the range and travel time of ray which is the time taken by the acoustic ray for sending the messages from anchor to sensor node to apply on different ray paths in a multi-layer medium. For this, initially, we choose the random launching angle θ of the ray. By using Snell’s law and sound velocity profile, we can calculate the incident angle for the next layer as the ray travels from one layer to another. The travel time and distance traveled by the ray will be calculated with the help of Figure 2 as,

(6) $$dr=\frac{dz}{tan\theta }$$

(7) $$ds=\frac{dz}{sin\theta }=\frac{dr}{cos\theta }$$

(8) $$dt=\frac{ds}{c\left(z\right)}$$

Now taking Equation (6):

$$r_{2}-r_{1}=\int_{z_{1}}^{z_{2}}\frac{dz}{tan\theta \left(z\right)}=\int_{z_{1}}^{z_{2}}\frac{cos\theta \left(z\right)dz}{\sqrt{1-cos^{2}\theta \left(z\right)}}$$

(9) $$r_{2}-r_{1}=\int_{z_{1}}^{z_{2}}\frac{\xi c\left(z\right)dz}{\sqrt{1-\xi^{2}c^{2}\left(z\right)}}$$

Similarly, now taking Equation (8):

(10) $$t_{2}-t_{1}=\int_{s_{1}}^{s_{2}}\frac{ds}{c\left(z\right)}=\int_{z_{1}}^{z_{2}}\frac{dz}{c\left(z\right)sin\theta \left(z\right)}$$

(11) $$t_{2}-t_{1}=\int_{z_{1}}^{z_{2}}\frac{dz}{c\left(z\right)\sqrt{1-\xi^{2}c^{2}\left(z\right)}}$$

“Because of the validity of these integrals on monotonic sections of the ray path, they are implemented on a point to point basis by placing points at the anchor, target sensor, layer boundary crossing (where gradient changes) and the point of inflections (where the sign of Δz changes)” [33].

When moving from point *i* $\left(r_{i},z_{i}\right)$ to point *i* + 1 $\left(r_{i+1},z_{i+1}\right)$ where $z_{i+1}-z_{i}<0$ (downwards), By using Equations (9) and (10), the horizontal distance covered by the ray will be:

(12) $$r_{i+1}-r_{i}=\frac{1}{\xi g_{i}}\left[\sqrt{1-\xi^{2}c^{2}\left(z_{i}\right)}-\sqrt{1-\xi^{2}c^{2}\left(z_{i+1}\right)}\right]$$

While the time of flight between these two points will be:

(13) $$t_{i+1}-t_{i}=\frac{1}{\left|g_{i}\right|}ln\left(\frac{c\left(z_{i+1}\right)}{c\left(z_{i}\right)}\;\frac{1+\sqrt{1-\xi^{2}c^{2}\left(z_{i}\right)}}{1+\sqrt{1-\xi^{2}c^{2}\left(z_{i+1}\right)}}\right)$$

However, when $z_{i+1}-z_{i}>0$ (upwards), the horizontal distance will be:

(14) $$r_{i+1}-r_{i}=\frac{1}{\xi g_{i}}\left[\sqrt{1-\xi^{2}c^{2}\left(z_{i+1}\right)}-\sqrt{1-\xi^{2}c^{2}\left(z_{i}\right)}\right]$$

Then time of flight will be:

(15) $$t_{i+1}-t_{i}=\frac{1}{\left|g_{i}\right|}\ln\left(\frac{c\left(z_{i}\right)}{c\left(z_{i+1}\right)}\;\frac{1+\sqrt{1-\varepsilon^{2}c^{2}\left(z_{i+1}\right)}}{1+\sqrt{1-\varepsilon^{2}c^{2}\left(z_{i}\right)}}\right)$$

Equation (12), can be write as,

(16) $$\Delta r_{1}=r_{1}-r_{0}=\frac{c_{o}}{cos\theta_{0}g_{o}}\left[\sqrt{1-cos^{2}\theta_{0}}-\sqrt{1-cos^{2}\theta_{0}\frac{c_{1}{}^{2}}{c_{o}{}^{2}}}\right]$$

Similarly,

(17) $$\Delta r_{2}=r-r_{1}=\frac{c_{1}}{cos\theta_{1}g_{1}}\left[\sqrt{1-cos^{2}\theta_{1}}-\sqrt{1-cos^{2}\theta_{1}\frac{c_{2}{}^{2}}{c_{1}{}^{2}}}\right]$$

By using the above Equations (16) and (17) we can calculate the total horizontal distance traveled by the ray which equals to $\Delta r_{1}+\Delta r_{2}+\Delta r_{3}+\ldots \Delta r_{n}$. If it is not close to the sensor then we repeat the whole procedure by taking different angles to make the ray traveled as much as close to the sensor.

### 5.3. Calculation of Direct Distance Traveled by the Ray

For accuracy, we will take the layer at a distance of 1 m that is why Pythagoras theorem is used for calculating the direct distance traveled by the ray $d_{i}$ from anchor to the sensor as,

(18) $$d_{s_{i}}=\sqrt{1^{2}+r_{i}^{2}}$$

After getting the value of $d_{s_{i}}$ from (18) we can be able to calculate the travel time by using Equation (10) as mentioned before since we are taking the depth of 1 m, therefore rather using Equation (11). In modified form (10) will give the time by using $d_{s_{i}}$ for each layer with the help of Equation (19),

(19) $$t_{i}=\frac{d_{s_{i}}}{v_{i-1}}$$

if the depth of each layer is greater than 1 than the travel time can be calculated by using Equations (14) and (15) in the form of,

(20) $$t_{i+1}-t_{i}=\frac{1}{\left|g_{i}\right|}ln\left[\frac{c_{i+1}}{c_{i}}\frac{1+\;\sqrt{1-\varepsilon^{2}c^{2}\left(z_{i}\right)}}{1+\;\sqrt{1-\varepsilon^{2}c^{2}\left(z_{i+1}\right)}}\right]$$

which is equals to

(21) $$t_{1}-t_{0}=\frac{1}{\left|g_{0}\right|}ln\left[\frac{c_{1}}{c_{0}}\frac{1+\;\sqrt{1-cos\theta_{0}{}^{2}}}{1+\;\sqrt{1-cos^{2}\theta_{0}\frac{c_{1}{}^{2}}{c_{o}{}^{2}}}}\right]$$

Then, each travel time will be added and divided by the corresponding summation of horizontal distance to get the accurate velocity, from anchor to sensor by using Equation (22),

(22) $$v_{equ}=\frac{d_{tot}}{t_{tot}}$$

As proposed, the study jointly estimating the energy-efficient time synchronization and location of underwater wireless sensors which have special importance and requirement in underwater wireless sensor networks (UWSNs). According to our methodology, this whole process will work within 5 phases which are as follows: phase I is the message exchange procedure, phase II is the calculation of sound velocity, phase III is the calculation of clock skew, phase IV is the calculation of position, phase V is the estimation of offset. According to the procedure the message exchange procedure in phase I, is a one-way from anchor to a sensor which performs a pair-wise synchronization mechanism for estimating the clock skew and offset. Due to the one-way broadcast of message packets, the proposed algorithm is energy efficient for synchronization and localization as the target sensors are silent and only receive signals from the anchor.

## 6. Pairwise Synchronization and Localization

We are considering the three-dimensional network with four anchor nodes. We assume that the anchor nodes are synchronized, and locations are known to calculate the ordinary sensor node’s skew, offset, and location.

The ordinary sensors clock is:

(23) T=αη+β

In the above Equation (23), *α* and *β* are the clock skew and clock offset respectively whereas *η* is the reference time and *T* is the time of the sensor node. 

As defined earlier in phase I the anchor nodes will broadcast one-way timestamps packets until the sensor node has got enough information about the timestamps. After receiving enough packets from all anchor nodes, initially, the ordinary sensor node will perform the calculation of propagation delay along with the calculation of velocity as described above. Propagation delay, between anchor nodes and ordinary sensor node, will be estimated in the first phase, which is further used as input for the next phase.

In the second phase, we compute clock skew by using the linear regression on the difference between the received timestamp and the time stamp in the packets. These packets only contain the time stamp information of the first phase. As defined earlier, in underwater communication, propagation is generated due to the low signal propagation, by using linear regression all the above-mentioned parameters can write as,

(24) $$T_{i}=\alpha \;\left(\eta_{i}+t_{i}\right)+\beta ,\;i\in \;\left\{1,2,\ldots \;\ldots ,\;N\right\}$$

where $T_{i}$ is the time of the sensor node B (node that needs to be synchronized and localized), $\eta_{i}$ and $t_{i}$ are the reference time and the propagation delay of *i*th anchor node respectively in Equation (24). Note that we required sufficient packets for the accurate calculation of skew, as the error of skew will affect the accuracy of our algorithm. To simplify the linear regression of skew, we have:

(25) $$\varepsilon_{i}^{j}≜\;\alpha t_{i}^{j}+\beta ,\;j\in \left\{1,2,\ldots \;\ldots ,\;N\right\}$$

Equation (25) shows the for *j*th packets of *i*th anchor node, where ε_*i*_ is the difference of clock skew. By subtracting Equations (24) and (25) we get:

(26) $$T_{i}^{j}=\alpha \eta_{i}^{j}+\varepsilon_{i}^{j},\;j\in \left\{1,2,\ldots \;\ldots ,\;N\right\}$$

For solving Equation (26) from linear regression we consider,

(27) P=Q∗M

(28) $$P={\left[T_{i}^{1},\;T_{i}^{2},\;\ldots \;\ldots ,\;T_{i}^{N}\right]}^{T},M={\left[\alpha ,\varepsilon \right]}^{T}$$

$$Q=\left[\begin{array}{cc} \eta_{i}^{1} & 1\\ \vdots & \vdots \\ \eta_{i}^{N} & 1 \end{array}\right]$$

We assumed the skew is unchanged in the received only synchronization mechanism. For minimizing the error, we are using the equations as follows:

*min* {*E*} = *min* {*e^T^ e*} = *min* {[*QM* − *P*] *^T^* [*QM* − *P*]} (29)

(30) $$\frac{\partial }{\partial M}{\left[QM-P\right]}^{T}\left[QM-P\right]=0$$

In the above Equation (29) *E* is the least square error of the estimation (LS estimator). Equation (30) can estimate *M*, also *M* (α,ε) can be calculated by (20),

*M* = (*Q^T^ Q*)^−1^ (*Q^T^P*) (31)

Our next step is to calculate the error due to clock offset to obtain more accuracy of the ordinary sensor node and its position as well. As the local time of sensor node contains both skew and offset error. Now by using the known locations of different anchors and the time with the help of time, we carry out our above linear regression equation for the position and the offset of the ordinary sensor node.

(32) $$T_{i}=\alpha \;\left(\eta_{i}+\frac{\gamma_{i}}{\nu }\right)+\beta$$

(33) $$\gamma_{i}=\left(\frac{T_{i}-\beta }{\alpha }-\eta_{i}\right)\nu =∥P_{A}-P_{S}∥$$

In the above Equation (32) $\gamma_{i}$ is the distance between anchor A and the ordinary sensor node. $P_{A}$ and $P_{S}$ is the position of anchor node and ordinary sensor node respectively, also the coordinates of a sensor node can be expressed as $[x^{s},y^{s},z^{s}]$ and *v* is the signal propagation speed in underwater acoustic channels. Here we have the depth information of both sensor and anchor node which are *z_i_* and *z* respectively. Also, we have the information on the location, and time of the anchor node. By putting the value of the distance formula in Equation (33), we get Equation (34) as,

(34) $$\left(\frac{T_{i}-\beta }{\alpha }-\eta_{i}\right)\nu =\sqrt{{\left(x_{i}-x^{s}\right)}^{2}+{\left(y_{i}-y^{s}\right)}^{2}+{\left(z_{i}-z^{s}\right)}^{2}\;},\;i\in \left\{\;1,\;2,\;\ldots \;\ldots ,\;N\right\}$$

For simplifying above equation, we use (35) and (36) will become,

(35) $$T_{i}^{\prime }≜\frac{T_{i}}{\alpha }-\eta_{i},\;i\in \left\{1,2,\ldots \;\ldots ,\;N\right\},\;\beta^{\prime }≜\frac{\beta }{\alpha }$$

(36) $$\sqrt{{\left(x_{i}-x^{s}\right)}^{2}+{\left(y_{i}-y^{s}\right)}^{2}+{\left(z_{i}-z^{s}\right)}^{2}}=\left(T_{i}^{\prime }-\beta^{\prime }\right)\nu$$

*α* has been obtained in the second phase. By using *α*, we can find $T_{i}^{j}$. However, *x*, *y*, and $\beta^{\prime }$ are not known to us in the above Equation (36). For the estimation of *x*, *y*, and $\beta^{\prime }$, we must have the information of at least three other anchor nodes to get three equations. Linear least square will be used for estimation. Linear least square solution is given by Equation (37),

(37) $$L*S=U\;where,\;S=\left[x,y,\beta^{\prime }\right]$$

(38) $$L=\left[\begin{array}{c} \begin{array}{ccc} 2\left(x_{2}-x_{1}\right) & 2\left(y_{2}-y_{1}\right) & \;\;2\left(T_{2}^{\prime }-T_{1}^{\prime }\right)\nu^{2}\\ 2(x_{3}-x_{1)} & 2\left(y_{3}-y_{1}\right) & \;\;\;2\;\left(T_{3}^{\prime }-T_{1}^{\prime }\right)\nu^{2}\\ \vdots & \vdots & \vdots \end{array}\\ \begin{array}{ccc} 2\left(x_{k}\;-\;x_{1}\right) & 2\;\left(y_{k}\;-\;y_{1}\right) & 2\;\left(T_{k}^{\prime }\;-\;T_{1}^{\prime }\right)\nu^{2} \end{array} \end{array}\right]\in \;C^{3\times 3}$$

(39) $$U=[\begin{array}{c} x_{2}^{2}-x_{1}^{2}+y_{2}^{2}-y_{1}^{2}+z_{2}^{2}-z_{1}^{2}-2\left(z_{2}-z_{1}\right)z+\left(T_{2}^{\prime }-T_{1}^{\prime }\right)\nu^{2}\\ x_{3}^{2}-x_{1}^{2}+y_{3}^{2}-y_{1}^{2}+z_{3}^{2}-z_{1}^{2}-2\;\left(z_{3}-z_{1}\right)z+\left(T_{3}^{\prime 2}-T_{1}^{\prime 2}\right)\nu^{2}\\ \vdots \\ x_{k}^{2}-x_{1}^{2}\;+\;y_{k}^{2}-y_{1}^{2}\;+\;z_{k}^{2}-z_{1}^{2}-2\;\left(z_{k}-z_{1}\right)z+\left(T_{k}^{\prime }{}^{2}-T_{1}^{\prime }{}^{2}\right)\nu^{2} \end{array}]$$

According to Equations (38) and (39) the ordinary sensor node can receive packets from different numbers of anchor nodes for the estimation of its location. Equation (40) estimates the error by using,

(40) $$min\left\{e^{T}\;e\right\}\;=\;min\{{\left[LS\;-\;U\right]}^{T}\;\left[LS\;-\;U\right]\}$$

(41) $$\frac{\partial }{\partial S}{\left[\;LS\;-\;U\right]}^{T}\;\left[LS\;-\;U\right]\;=\;0^{\;}$$

As indicated by the Equation (41), we can find out *S*, or use the Equation below to calculate *S*.

(42) $$L^{T}\;LS=L^{T}\;U$$

(43) $$S={\left(L^{T}\;L\right)}^{-1}L^{T}\;U$$

With the help of (43), *x*, *y*, and $\beta^{\prime }$ have now known us. By putting $\beta^{\prime }$ in Equation (35), the offset will be obtained. By using linear regression twice in the first and second phases of our algorithm, the ordinary sensor node can be synchronized and localized.

### 6.1. Iterations

The output of the second phase, the sound speed will be fed into the third phase which calculates the skew which is used as an input to find the location coordinates of sensors in the fourth phase, and in the fifth or last phase, we find the offset. The process of iteration will continue until the errors of skew, offset and location, decrease, and converge to a constant value.

### 6.2. Summary of E^2^JLS

All the operations of each phase will be summarized in this section, as indicated earlier that the proposed algorithm is based on five phases. Phase I is the message exchange procedure between anchor to sensor node that need to be synchronized and localized. Phase II calculates the time taken by the acoustic ray by using the velocity of sound and the distance traveled by the acoustic ray. The acoustic ray is used to send the messages for the localization and synchronization of sensor nodes. This phase actually calculates the propagation delay, furthermore, phase III gives the calculation of skew and phase four gives the location of an unknown sensor node. Phase five provides the offset. This is an iterative procedure that gradually improves accuracy.

## 7. Calculation of Cramer Rao Lower Bound for the Proposed Algorithm

The proposed $E^{2}JSL$ use least square estimation, which is an unbiased estimator, which means that the Cramer-Rao lower bound can be derived from the LS estimator [6]. To calculate the CRLB for $E^{2}JSL$ method we provide the performance reference as, 

Suppose $V=\left[\eta_{A},\;T_{A},{\;\eta }_{B},\;T_{B}\right]$ is a vector of measurement with mean μ(ϕ) and covariance C. Also ϕ is defined as the measurement of timestamps of ordinary node location x, y and clock parameters α, β represented as,

(44) $$\mu \left(\phi \right)={\left[\alpha \eta_{1,1}+\beta ,\;\ldots \;\ldots \;\ldots \;\ldots \;\ldots \;\alpha \eta_{1,n}+\beta \right]}^{T}$$

or Equation (33) can be written as,

(45) $$\mu \left(\phi \right)=\left[\frac{T_{2,1}^{1}-\beta }{\alpha }-t_{1},\;\ldots \;\ldots \;\ldots \;\ldots \;\frac{T_{2,n}^{n}-\beta }{\alpha }-t_{n}\right]$$

where *C* $=\sigma^{2}$ denotes the F × F identity matrix. The Fisher matrix can be represented as,

(46) $$J\left(\phi \right)={\left[\frac{\partial \mu }{\partial \emptyset }\right]}_{i}^{T}C^{-1}{\left[\frac{\partial \mu }{\partial \emptyset }\right]}_{j}^{T},i,j=1,2,3,4,5$$

where *i*, *j* stands for (*i*, *j*) element of the matrix, ${\left[.\right]}_{i}$ stands for *i*th element of the vector.

$${\left[\frac{\partial \mu }{\partial \emptyset }\right]}_{i}=\{\begin{array}{c} \frac{\partial \mu }{\partial x}\;if\;i=1\\ \frac{\partial \mu }{\partial y}\;if\;i=2\\ \frac{\partial \mu }{\partial z}\;if\;i=3\\ \frac{\partial \mu }{\partial \alpha }\;if\;i=4\\ \frac{\partial \mu }{\partial \beta }\;if\;i=5 \end{array}$$

Similarly,

$$\frac{\partial C}{\partial \emptyset }=\;\{\begin{array}{c} dia\sigma^{2},\;\ldots \;\ldots \sigma^{2},\;if\;i=4\\ 0\;otherwise \end{array}$$

The partial derivative ${\left[\frac{\partial \mu }{\partial \emptyset }\right]}^{T}=\left[\frac{x-x^{s}}{c\gamma_{1}}-\frac{y-y^{s}}{c\gamma_{1}}-\;\frac{T_{2,\;1}-\beta }{\alpha^{2}}-\frac{1}{\alpha },\;\frac{x-x^{s}}{c\gamma_{2}}-\frac{y-y^{s}}{c\gamma_{2}}-\;\frac{T_{2,\;2}-\beta }{\alpha^{2}}-\frac{1}{\alpha }\ldots \;\ldots \right]≜K$ where $\gamma_{i}=\sqrt{{\left(x-x^{s}\right)}^{2}+{\left(y-y^{s}\right)}^{2}+{\left(z-z^{s}\right)}^{2}}$

$${\left[J^{-1}\right]}_{i,j}=\sigma^{2}{\left(K^{T}K\right)}^{-1}$$

co-variance *C* = $\sigma^{2}I$, therefore,

The lower bound on the error variance of any unbiased estimator for the position and the clock parameter can be calculated as,

$$E\;∥\hat{x}-x{∥}^{2}\geq \sum_{i=1}^{3}{\left[J^{-1}\right]}_{i,j}$$

$$E\;∥\hat{\alpha }-\alpha {∥}^{2}\geq {\left[J^{-1}\right]}_{4,4}$$

$$E\;∥\hat{\beta }-\beta {∥}^{2}\geq {\left[J^{-1}\right]}_{5,5}$$

### 7.1. Computational Complexity Analysis

Computational complexity will be discussed in this section which is based on the total number of flops of the considered estimator in the proposed study. Here we assume addition subtraction and multiplication operation can be computed as one flop in the real domain [19]. Whereas division or square root operation is considered as 20–30 flops. The total number of flops will be evaluated for the proposed method to analyze the complexity as the growth of the approach.

In phase II the computation of propagation delay requires 3N(K + 1) multiplications, 3NK + 2N addition, and NK divisions. In contrast, the construction of L requires 6NK + N multiplications, N(K + 1) addition, and NK division. In Equation (32) we separately calculate $L^{T}L$ and $L^{T}$ and multiply these two calculations. The overall computational complexity will be 18NK + 20 multiplication and 20NK addition and 3-D matrix inversion. Similarly, the overall complexity of Phase III includes 28N(K + 1) + 31 multiplications, 30N(K + 1) addition 2N(K + 1) division and N(K + 1) root operation with 4-D matrix inversion [15]. Based on the calculation, we can say that with the number of observations the overall complexity increases linearly. The order of complexity of [15] is the same but, due to the presence of a greater number of unknowns, it shows more complexity as compared to the proposed technique.

The average running time of different algorithms has been compared on the basis of computation of complexity. Considering the network based on 8 anchor nodes we performed all the simulations on MATLAB by initializing the location and clock parameter values. The algorithms have been run for 1000 realizations of the considered network and compute the average running time. From Table 1, it can see that the proposed technique gives low running time in (ms) as compared to other approaches which also leads to the conclusion that *E*^2^*JSL* gives reasonable complexity.

## 8. Simulation Settings

**Figure 3:**
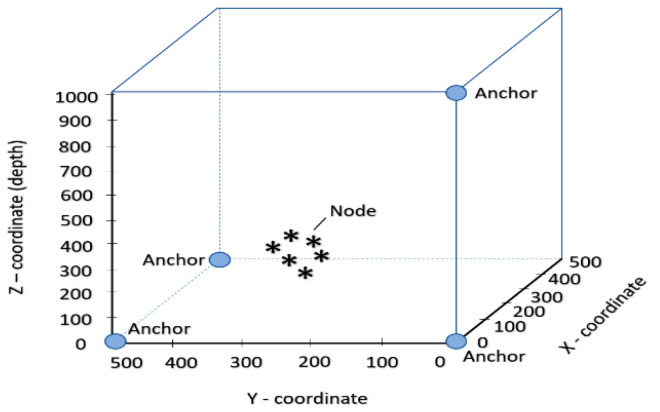
Dimension of nodes having the area of 500 m × 500 m × 1000 m with randomly placed anchor and sensor.

In this study, all the simulations have been done in MATLAB. The proposed Simulation architecture consists of four or more anchor nodes and one ordinary node which is located at d meter away from the cube center of gravity. For the implementation, we have considered the area of 500 m × 500 m × 1000 m region and here we have randomly placed an ordinary sensor node with 300 m of depth as given by Figure 3. We have used this dimension for the comparison with other methods, based on practical consideration. All anchors, as well as the sensor nodes of underwater, are commercials, based on high quality and good fairness. Note that the performance of the proposed methodology will not be affected by changing the dimension of simulation. Figure 3 shows the geometry and location of the ordinary sensor node and anchor nodes. Gaussian distributed random skew and offset relative to the clock of anchor nodes with 1 and 0 s as the mean values and 0.001 and 0.5 s^2^ as the variances, respectively. For the simulation of time synchronization error, initial skew and clock offset are considered as $\Delta_{thresh}$ = 15 and the initial time slot was set to be $T_{slotdur}$ = 5 s. Initial sound speed at the surface is b m = 1420 m/s with environment constant a and average sound speed is taken as c = 1500 m/s. To prove the validity of the proposed algorithm we have made a comparison with some previously developed techniques such as STSL, JSL, and GNJSL since all of them are joint localization and time synchronization techniques. In the simulation setting, we have used 20 number of messages, where $t_{all}$ is taken to be 106 s, 40 bytes $l_{p}$ is taken for both JSL and STSL as they employ a two-way message exchange and 25 $l_{p}$ is set for both GN-JSL [19] and $E^{2}JSL$ as both of them employ one-way messaging approach. The performance of the proposed algorithm is evaluated on the basis of synchronization accuracy, localization accuracy, and energy efficiency by calculating some parameters such as propagation delay measurement noise, the number of messages, and the number of reference nodes that directly affect these algorithms. We conduct Monte Carlo (MC) simulations, and all simulation results are averaged of $N_{mc}$ = 2000 independent runs.

## 9. Performance Evaluators

To measure the performance of the proposed algorithm we use three evaluators which are RMSE of the location of the ordinary node and clock parameters, which are skew and offsets. The third evaluator is energy efficiency. RMSE of location and clock parameters are defined as,

(47) $$RMSE_{L}=\sqrt{\frac{\sum_{i=1}^{N_{mc}}({\left(x-\hat{x}\right)}^{2}+{\left(y-\hat{y}\right)}^{2}+{\left(z-\hat{z}\right)}^{2}}{N_{mc}}}$$

(48) $$RMSE_{s}=\sqrt{\frac{\sum_{i=1}^{N_{mc}}{\left(\varrho -\hat{\varrho }\right)}^{2}}{N_{mc}}}$$

where ϱ=α=β and $\hat{\varrho }=\hat{\alpha }=\hat{\beta }$.

In the above Equation (36) $\hat{x},\;\hat{y}\;\mathrm{and}\;\hat{z}$ are the estimated coordinates of ordinary sensors node. Similarly, $\hat{\varrho }$ are the corresponding estimated skew and offset of that node in Equation (37). Also, the energy efficiency can be calculated as,

(49) $$\varsigma =\frac{t_{all}}{k_{s}n_{m}l_{p}}$$

where $n_{m}$ is the number of messages per execution $k_{s}$ and $l_{p}$ is the packet size and $t_{all}$ is the time in seconds in Equation (38) which is used to keep the clock error below a certain value.

### 9.1. Relation between Number of Messages and Estimation Errors

**Figure 4:**
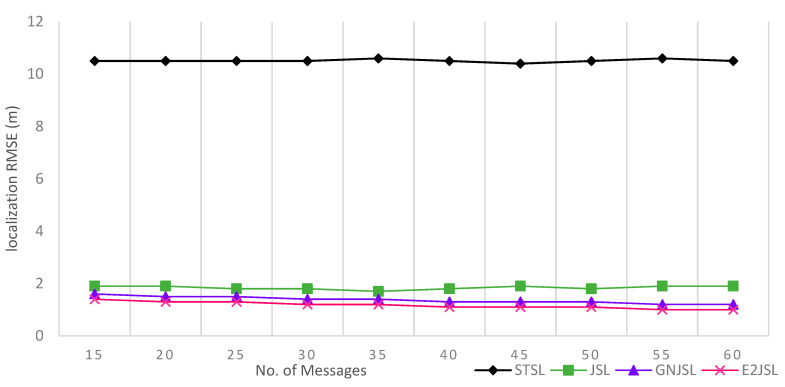
Relationship between number of messages and localization error RMSE (m).

**Figure 5:**
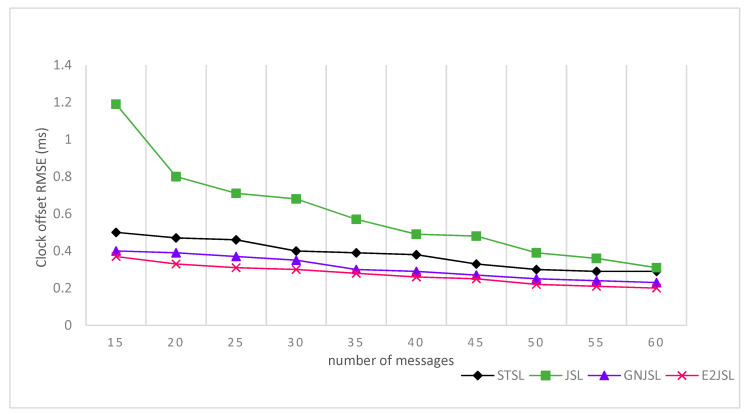
Relationship between a number of messages clock offset RMSE (ms).

**Figure 6:**
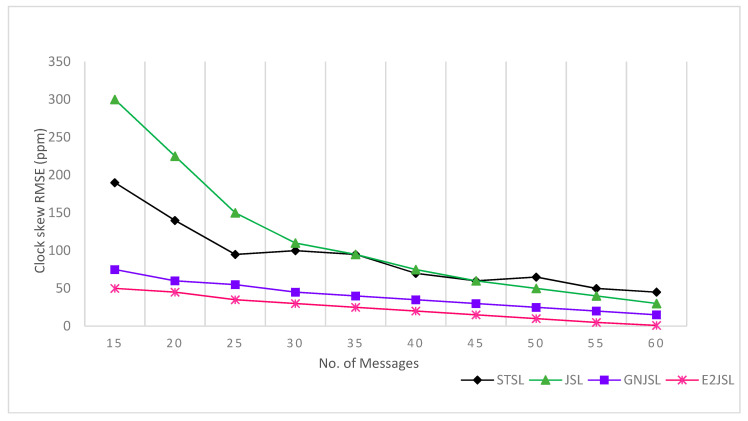
Relationship between number of messages and clock skew RMSE (ppm).

Figure 4, Figure 5 and Figure 6 indicate the relation between the number of messages and the estimation error for localization and clock parameters to analyze the performance of the proposed algorithm. In our simulations, the proposed algorithm has been executed a number of times ranging from 20 to 80 along with the algorithms discussed in state of art STSL, GN-JSL, and JSL. As a result of the implementation, we have seen that the overall performance of the proposed method is much better than other methods. The reason behind this performance is that both STSL and JSL use linear regression to estimate clock skew and offset and the accuracy of their calculation is based upon the number of message exchanges. Where GN-JSL is based upon the more accurate estimation of initial position and estimation of initial position is also based upon the large number of messages involved. However, as it is seen in Figure 4 that $E^{2}JSL$ localization accuracy is much better than the STSL [13] and JSL [11], because both these algorithms did not consider the stratification effect, thus increasing the number of messages can’t give an accurate estimation. Whereas the performance of GN-JSL [19] is slightly better than both these algorithms because of the consideration of the stratification effect. Similarly, the clock parameters accuracy estimated by $E^{2}JSL$ is better than other algorithms given by Figure 5 and Figure 6, JSL and STSL are almost unaffected by the number of messages. So, we can conclude that the proposed algorithm that initially estimates the stratification effect by using the ray-tracing approach performs well, and without consideration of the stratification effect, it is not possible to get the accuracy on both localization and synchronization.

### 9.2. Relation between the Number of Reference Nodes and Estimation Errors

**Figure 7:**
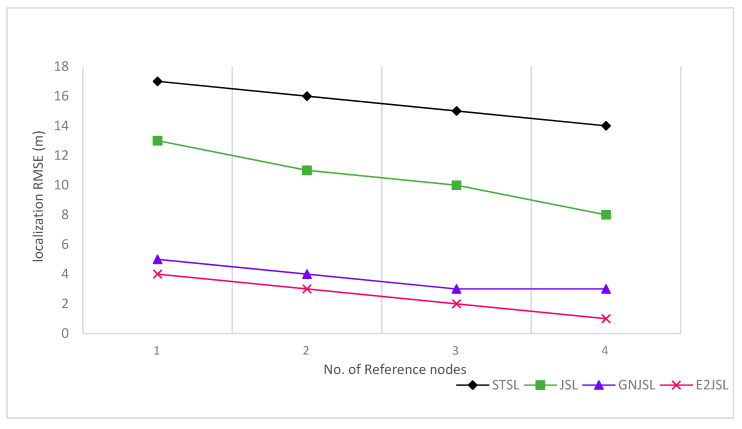
Relationship between the number of reference nodes and Localization RMSE (m).

**Figure 8:**
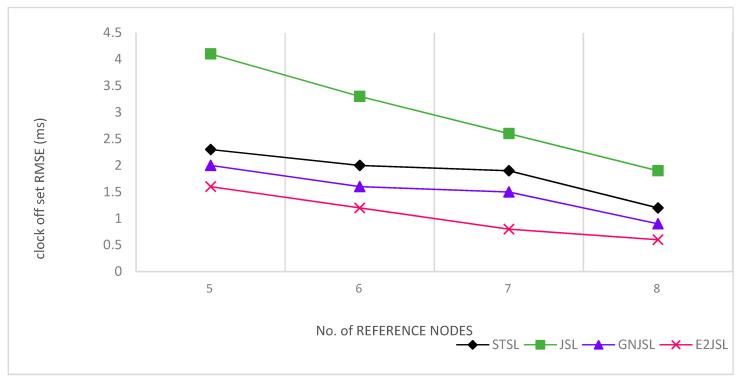
Relationship between the number of reference nodes and clock offset RMSE (ms).

**Figure 9:**
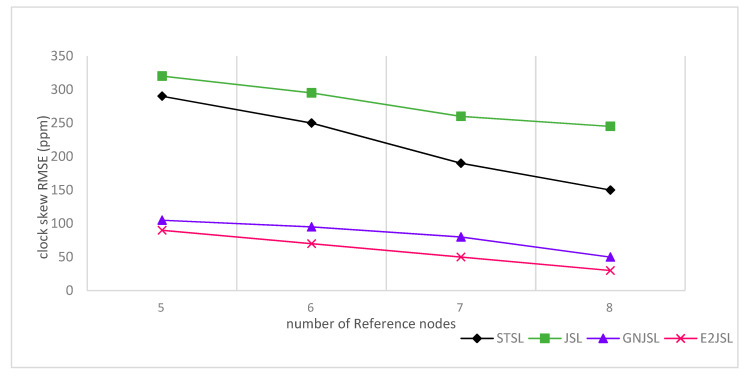
Relationship between number of reference node and clock skew RMSE (ppm).

In Figure 7, Figure 8 and Figure 9 the relation between the number of reference nodes and estimation error is given to see the performance of the proposed algorithm. In our simulation, we have added an anchor node one by one on the vertices of the cube as shown by the figures. Initially, we have taken 4 number of reference nodes and gradually increase these reference nodes up to 8. According to Figure 7, Figure 8 and Figure 9, it is clear that by increasing the number of reference nodes all the considered algorithms are getting better in terms of both localization and synchronization but $E^{2}JSL$ gives better accuracy as compared to other previously developed methods.

### 9.3. Energy Efficiency of Proposed Technique

**Figure 10:**
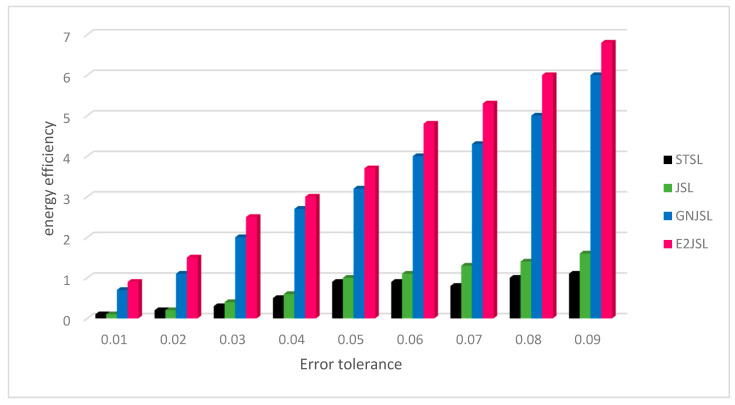
Relationship between error tolerance and energy efficiency.

Energy efficiency has always been a serious issue in traditional wireless sensor networks but as far as the underwater sensors networks are concerned it is even more difficult to maintain the energy level among the sensors due to many underwater-related issues which we have discussed earlier. Therefore, we have made the simulation based on energy efficiency and compare the energy consumed by each considered algorithm during the process of localization and synchronization. As compare to radio communications, acoustic modems consume more energy while transmitting and receiving the data. From Figure 10 it can be seen that STSL and JSL consume more energy as compare to GN-JSL and $E^{2}JSL$ the reason behind this, is that both STSL and JSL employ a two-way message exchange procedure that consumes more energy. On the other hand, in both GN-JSL and $E^{2}JSL$, reference nodes broadcast a one-way message for the procedure of localization and synchronization. This makes these algorithm procedures energies efficient. We have considered the energy efficiency according to the error tolerance ranging from 0.01 to 0.09 and it is clear from Figure 10 that energy efficiency of all considered algorithm increase with the relaxation of tolerance. These results show that the proposed algorithm has high energy efficiency as compared to all other algorithms for all values of tolerance error. It is concluded that $E^{2}JSL$ can able to reduce the message overhead which is the most basic requirement of acoustic sensors. As $E^{2}JSL$ shows high synchronization accuracy in comparison with other considered techniques.

### 9.4. The Relation between Number of Iterations and Estimation Errors

**Figure 11:**
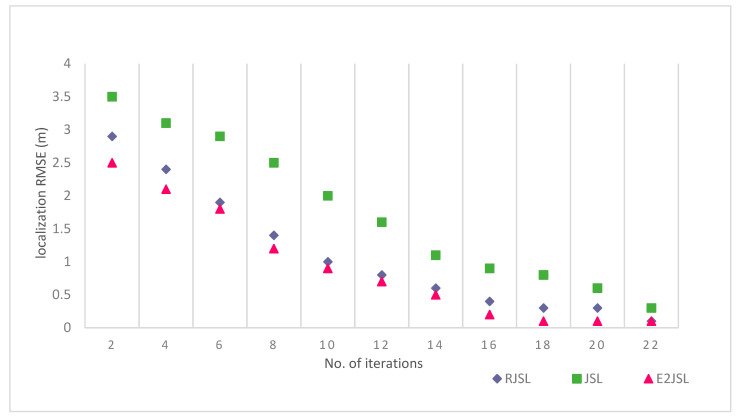
Relationship between number of iterations and Localization RMSE (m).

**Figure 12:**
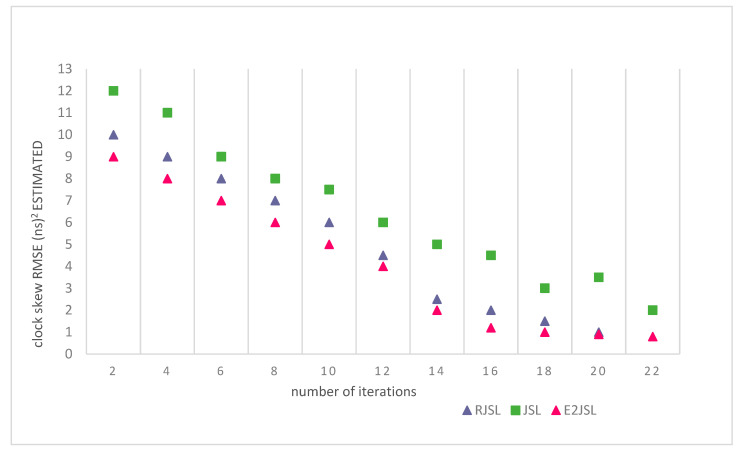
Relationship between number of iterations and clock skew.

**Figure 13:**
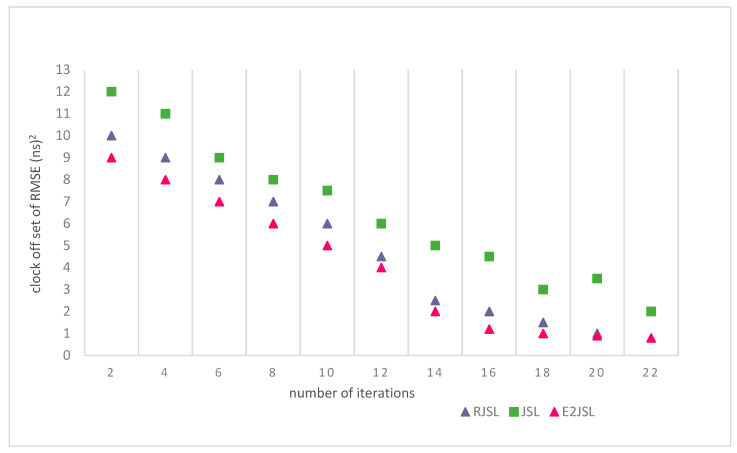
Relationship between number of iterations and clock offset RMSE (ns)^2^.

To evaluate the performance of the proposed algorithm, we have also done the simulation on the basis of the number of iterations to see the effect on the estimation error of localization and synchronization. We have made a comparison of the proposed algorithm with other techniques like RJSL [14] and TSLA [16]. According to the simulation setting, we execute our considered algorithm almost 20 times to analyze the stability in terms of location estimation errors and clock estimation errors. As a result of simulations, it is clear from the figure that localization and synchronization errors are gradually moving towards stability as the number of iterations increases. This also indicates that by the increase of the number of iterations the error of skew, offset and location decreases. As indicated by Figure 11, Figure 12 and Figure 13 we can say that a greater number of iterations can bring stability in location, clock skew, and errors respectively. It is also clear by the figures that after 15 iterations the change in the estimation error is almost negligible in RJLS this is due to the fact that RJLS calculates the propagation delay with the help of sensor position which gradually improves the sound speed and synchronization accuracy. However, the proposed algorithm archives its stability in location and synchronization errors after 12 iterations whereas the TSLA is not able to get the accuracy after 15 or 16 iterations. So, we conclude our discussion in a way that $E^{2}JSL$ gives more accuracy in terms of localization and time synchronization as compare to other considered algorithms.

### 9.5. Performance of $E^{2}JSL$ with CRLB

**Figure 14:**
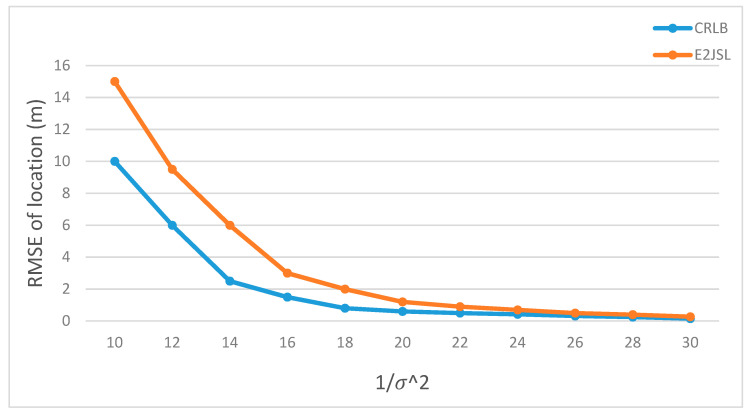
RMSE of location estimates vs. $1/\sigma^{2}e$.

**Figure 15:**
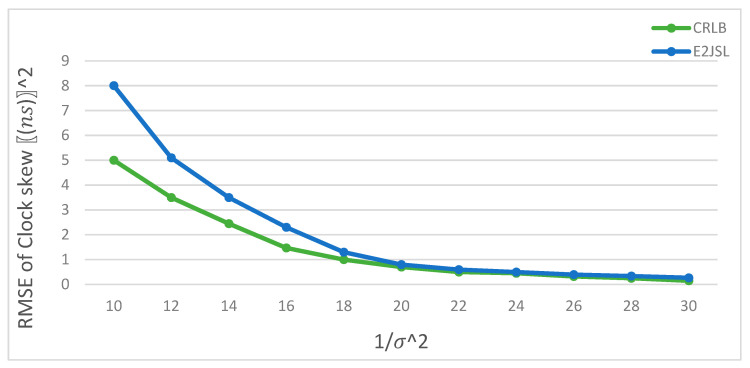
RMSE of clock skew estimates vs. $1/\sigma^{2}$.

**Figure 16:**
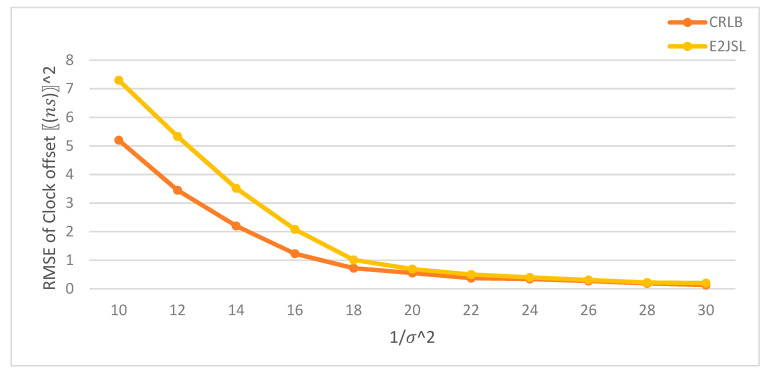
RMSE of clock offset estimates vs. $1/\sigma^{2}$.

We have compared the performance of the proposed $E^{2}JSL$ with CRLB. As mentioned earlier that we are considering the static anchor nodes which are already localized and synchronized and moving sensor node that needs to be localized and synchronized. It is given by Figure 14, Figure 15 and Figure 16 that the estimation error of location is taken as a function of $\frac{1}{\sigma^{2}}$ for the proposed method and CRLB. According to Figure 14, we can say that $E^{2}JSL$ proves to be more accurate in terms of location because its performance falls on the CRLB which is obvious. Similarly, the performance of $E^{2}JSL$ in comparing CRLB with estimation error of the clock parameter as a function of variance is sufficiently accurate according to Figure 15 for clock skew and Figure 16 for clock offset respectively. As both RMSE of clock parameters touches CRLB. So, we conclude that the proposed estimator achieves more accurate synchronization and location.

## 10. Conclusions

The proposed study has given an efficient approach to simultaneously synchronize and localize the underwater sensors that consumed the energy as low as possible. This is a low complexity five-phase algorithm for underwater sensor communication. The proposed methodology is based on a ray-tracing approach which helps in compensation of the stratification effect. The designed model is a one-way message exchange procedure and Snell’s law has been used to derive the relation for variation of sound speed which compensates the stratification effect. The design algorithm localized and synchronized a single ordinary sensor node with low energy consumption. The calculation of the Cramer-Rao lower bound (CRLB) proves the convergence of the proposed methodology. Error analysis has been given to prove the accuracy through simulation. CRLB has theoretically proved the accuracy of the proposed algorithm. Additionally, the computational complexity will increase with the number of observations.

Experimental results have proved that the provided algorithm outperforms the other previously developed results, our developed model has been compared in terms of several numbers of reference nodes with clock skew error, and offset error, also we have made the comparison between the number of reference nodes and localization an error (RMS). The results of our simulations show that the localization errors, clock skew and offset errors are more stable with the increase in number of reference nodes and number of messages. Energy efficiency has been compared with an error tolerance of different previous algorithms along with the proposed algorithm. All the presented results have proved that the provided methodology is sufficient for the localization and synchronization of underwater sensor networks as compared to other algorithms.

For future work, it may be suggested that the proposed algorithm can be extended for a multi-hop environment by making some assumptions to extend the localization coverage area. To the best of our knowledge and according to the literature survey, a joint procedure of localization and time synchronization for the multi-hop environment has not been given yet. Rather, they have been considered separately. Also, we have used a maximum of eight reference nodes, which can be extended in future work.

## Figures and Tables

**Table 1:** Average running time of different algorithms.

Algorithm	Average Time (ms)
GN-JSL	0.67
JSL	97.70
STSL	0.15
*E* ^2^ *JSL*	0.42
